# Supplementary material for: The 18S rRNA Methyltransferase DIMT-1 Regulates Lifespan in the Germline Later in Life
Source: Res Sq. 2024 Jun 21:rs.3.rs-4421268. Preprint. [Version 1] doi: 10.21203/rs.3.rs-4421268/v1 (PMC11213213; doi:10.21203/rs.3.rs-4421268/v1)
Supplement: 1 [file NIHPPRS4421268v1-supplement-1.pdf]

Extended Data Fig. 1. DIMT-1 knock-down affects endoplasmic reticulum unfolding protein response (UPR^{ER}). a, No difference in HSP-16.2 expression in response to *dimt-1* knock-down as assessed by GFP fluorescence 5 hours after 30 minutes of heat shock at 37°C in *Phsp-16.2::gfp* (zSi3000) (UPR^{cytosol}*)* worms. **b,** No difference in HSP-6 expression in response to *dimt-1* knock-down as assessed by GFP fluorescence after 5 hours of ethidium bromide (25 µg/mL) treatment in *Phsp-6::gfp* (zcIs13*)* (UPR^{mitochondria}*)* worms. **c,** Decrease in HSP-4 expression in response to *dimt-1* knock-down as assessed by GFP fluorescence after 5 hours of tunicamycin (5 µg/mL) treatment in *Phsp-4::gfp*(*zcIs4) (*UPR^{ER}*)* worms. All quantification was done using ImageJ software. Statistics represent unpaired t-test with Welch's correction. ns not significant, ** $p < 0.01$, **d,** Knock-down of *dimt-1* or mutation of the catalytic domain (E79A) caused increased survival on the ER stress inducer tunicamycin.

Extended Data Fig. 2. Ribosomal RNA methyltransferases and modifications are dynamically regulated throughout life and change in response to heat stress. a, Gene expression profile of putative rRNA methyltransferases in *C. elegans* from day 4 to day 19 age gradient. This data was generated by an analysis of gene expression data from[19]. **b,** *dimt-1* transcript levels increased with age relative to actin control as assessed by quantitative RT PCR. **c,** UHPLC-ms/ms analysis of rRNA modification levels in 26S and 18S rRNA subunits in an age gradient. Experiments were performed with 4 biological replicates. **d,** Gene expression profile of putative rRNA methyltransferases in *C. elegans* exposed to heat stress compared to control. This data was generated by an analysis of gene expression data from[7]. **e,** UHPLC-ms/ms analysis of rRNA modification levels in 26S and 18S rRNA subunits in worms exposed to heat stress compared to control. Experiments were performed with 4 biological replicates. **f,** UHPLC-ms/ms analysis of rRNA modification levels in 26S and 18S rRNA subunits in worms exposed to UV stress compared to control. Experiments were performed with 4 biological replicates. ns not significant, * $p < 0.05$, ** $p < 0.01$, *** $p < 0.001$, **** $p < 0.0001$.

Extended Data Fig. 3. Lifespan extension by DIMT-1 does not require *clk-1* and occurs in the germline. a, *dimt-1* knock-down increases in WT and *clk-1(e2519)* mutant worm lifespan to a similar extent (p=0.0855 by 2-way ANOVA). **b,** AID-induced DIMT-1 depletion extends lifespan in two different ubiquitous driver strains and when depleted specifically in the germline. **c,** *dimt-1* knock-down increases lifespan of EV484 mutant worms. Statistics and replicate experiments are presented in Supplementary Tables 1 and 2. ns; not-significant, *; $p < 0.05$, **; $p < 0.01$, ***; $p < 0.001$, ****; $p < 0.0001$ as calculated by log-rank (mantel-cox) statistical test.

Extended Data Fig. 4. *dimt-1* depletion causes a misregulation of expression of genes involved in longevity regulation, MTOR and FoxO/DAF-16 signaling and degradation pathways. a, A heat map of 5,765 differentially transcribed genes in response to *dimt-1* knock-down in day 7 worms. *dimt-1* was knocked down from the L4 stage until day 7. Each column represents an independent biological replicate. **b-c,** Pathway analysis of genes which show increased expression in response to *dimt-1* knock-down reveal genes involved in longevity regulation, TGF-β, WNT and MAPK signaling pathways, as well as degradation pathways including proteasome, peroxisome, and autophagy genes. **d-e,** Pathway analysis of genes which showed decreased expression in response to *dimt-1* knock-down reveal genes involved in protein processing, MTOR and FoxO signaling pathway, and ribosome biogenesis genes. Gene details and gene ontology pathways is presented in Supplementary Table 3.

Extended Data Fig. 5. *dimt-1* depletion causes altered binding of the ribosome to transcripts involved in longevity regulation, oxidative phosphorylation, degradation pathways, and glutathione metabolism in the germline of day 7 worms. a, Pathway analysis of 2082 differentially bound transcripts in the germline of day 7 worms treated with *dimt-1* RNAi from the L4 stage until day 7 reveals transcripts involved in longevity regulation, oxidative phosphorylation, degradation pathways and glutathione metabolism. **b-c,** Pathway analysis of transcripts that show increased binding in response to *dimt-1* depletion include genes involved in longevity regulation, fatty acid metabolism, glutathione metabolism, metabolism, and various degradation pathways. **d-e,** Pathway analysis of transcripts that show decreased binding in response to *dimt-1* depletion in the germline include genes involved in the ribosome, proteasome, oxidative phosphorylation and longevity regulation. Gene details and gene ontology pathways is presented in Supplementary Table 4.

Supplementary Table 1. Dimt-1 depletion extends lifespan in a raga-1 and germline dependent manner The figure panels in which specific experiments are shown or used are indicated in the right column. The mean lifespan and SD values were calculated by Prism from triplicate samples of 30 worms each (90 worms total). # worms: number of observed dead worms at the end of the experiment/number of alive worms at the beginning of the experiment. The difference between both numbers corresponds to the number of censored worms (worms that underwent "matricide", exhibited ruptured vulva, or crawled off the plates). P values are calculated by log rank (Mantel-Cox) statistical test.

Supplementary Table 2. DIMT-1 functions in the germline to regulate lifespan The figure panels in which specific experiments are shown or used are indicated in the right column. The mean lifespan and SD values were calculated by Prism from triplicate samples of 30 worms each (90 worms total). # worms: number of observed dead worms at the end of the experiment/number of alive worms at the beginning of the experiment. The difference between both numbers corresponds to the number of censored worms (worms that underwent "matricide", exhibited ruptured vulva, or crawled off the plates). P values are calculated by log rank (Mantel-Cox) statistical test.

Supplementary Table 5. DIMT-1 functions after mid-life to regulate lifespan The figure panels in which specific experiments are shown or used are indicated in the right column. The mean lifespan and SD values were calculated by Prism from triplicate samples of 30 worms each (90 worms total). # worms: number of observed dead worms at the end of the experiment/number of alive worms at the beginning of the experiment. The difference between both numbers corresponds to the number of censored worms (worms that underwent “matricide”, exhibited ruptured vulva, or crawled off the plates). P values are calculated by log rank (Mantel-Cox) statistical test.

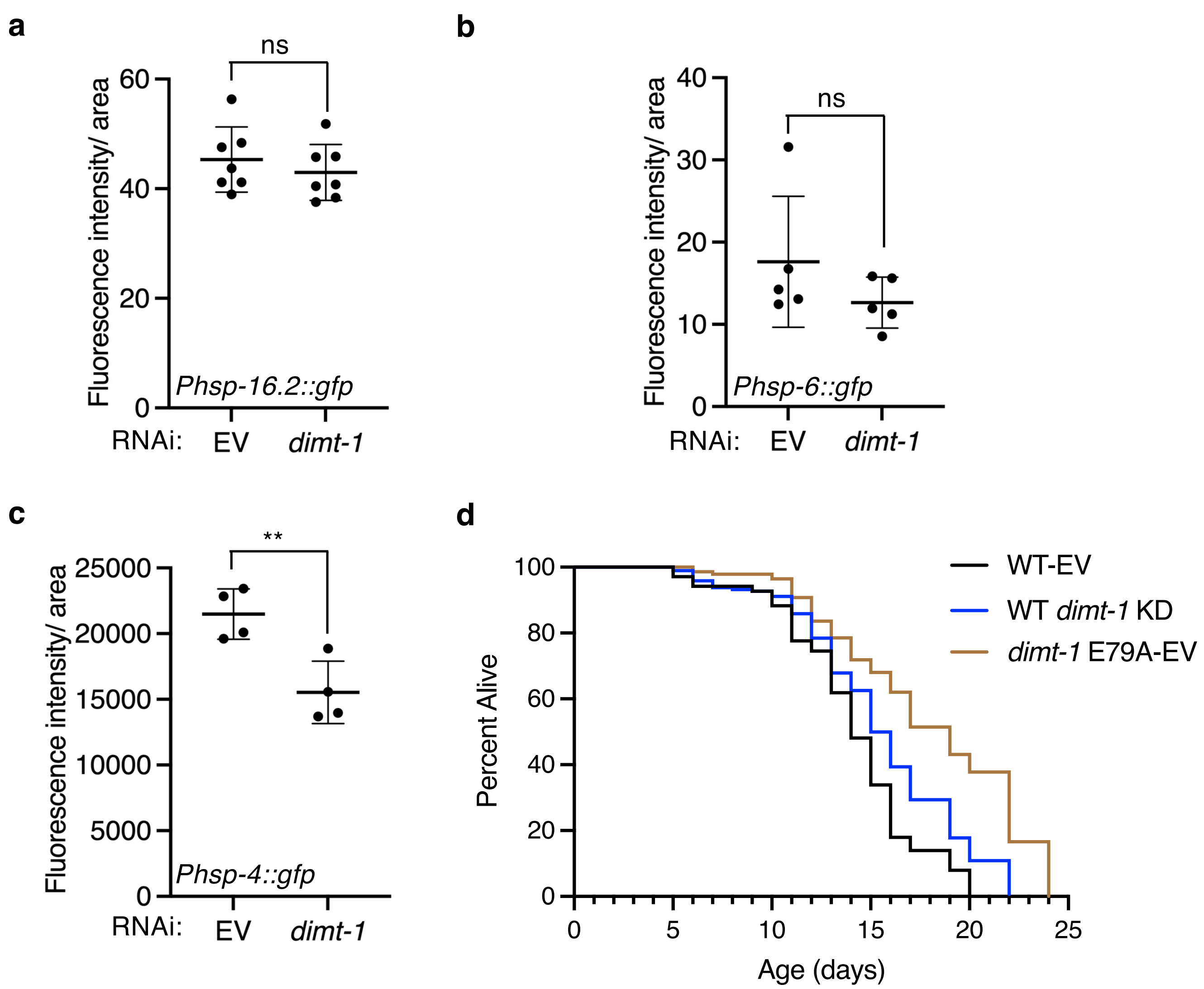

Rothi MH, Sarkar GC, Al Hadad J et al Fig S1

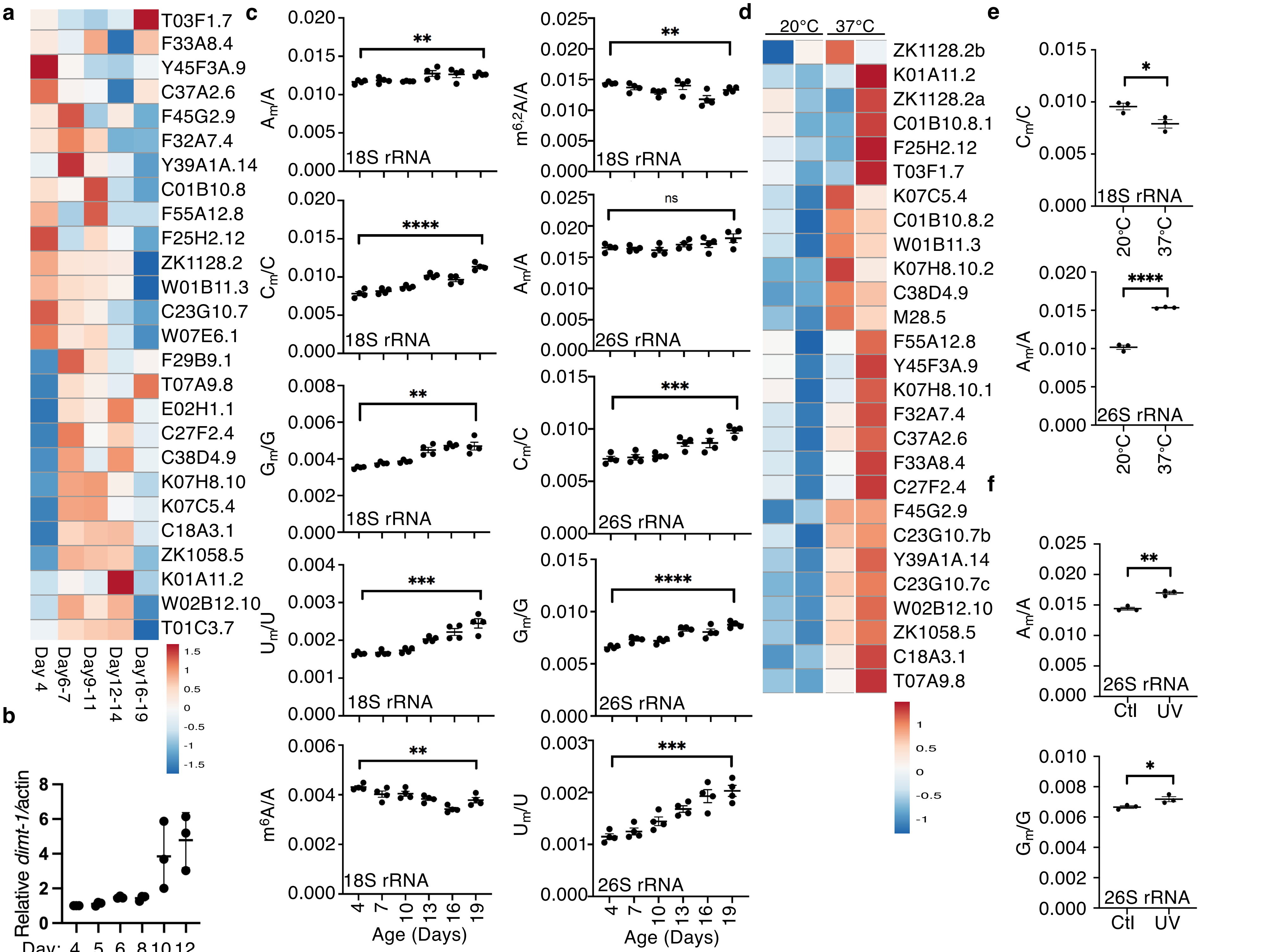

Rothi MH, Sarkar GC, Al Hadad J et al Fig S2

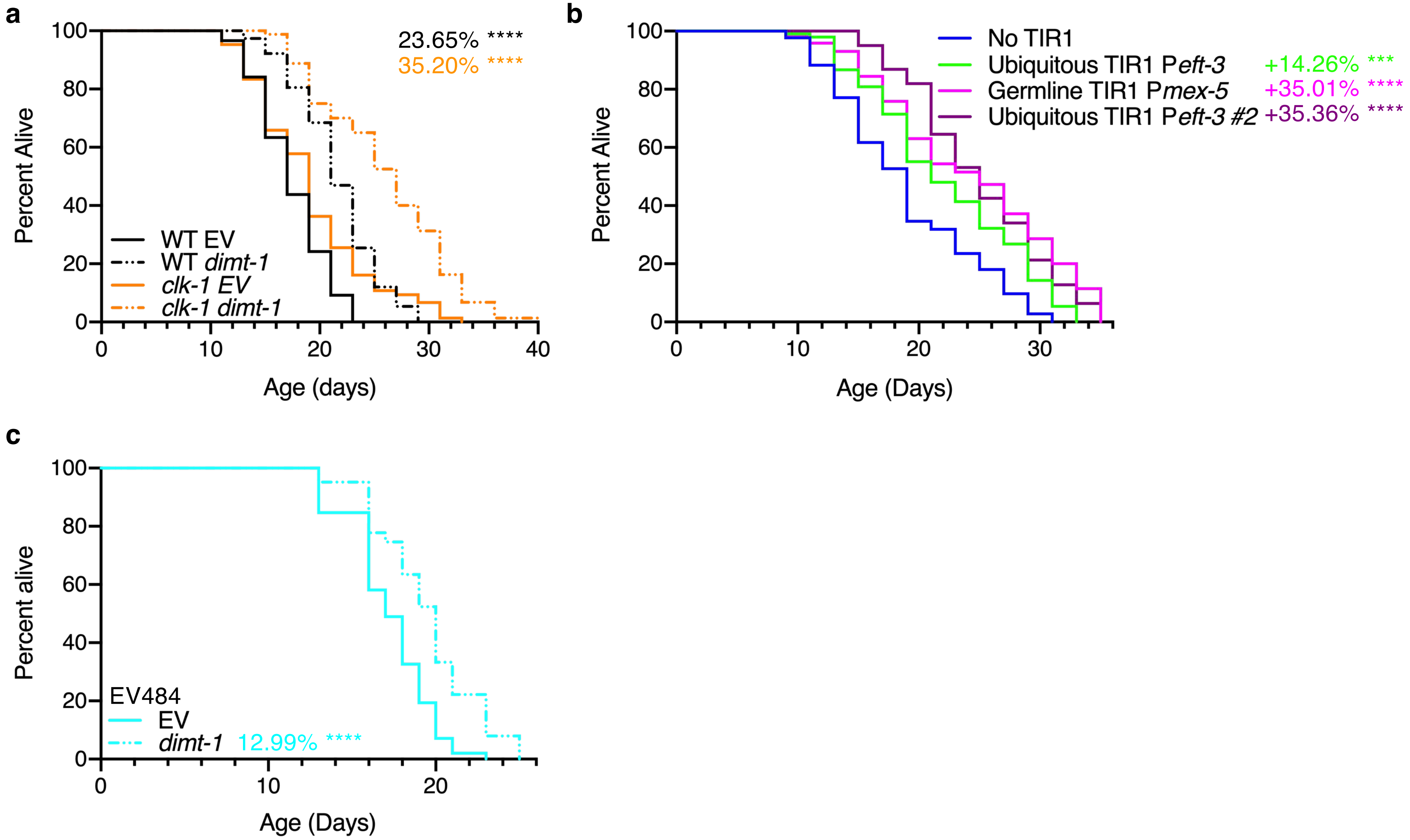

Rothi MH, Sarkar GC, Al Hadad J et al Fig S3

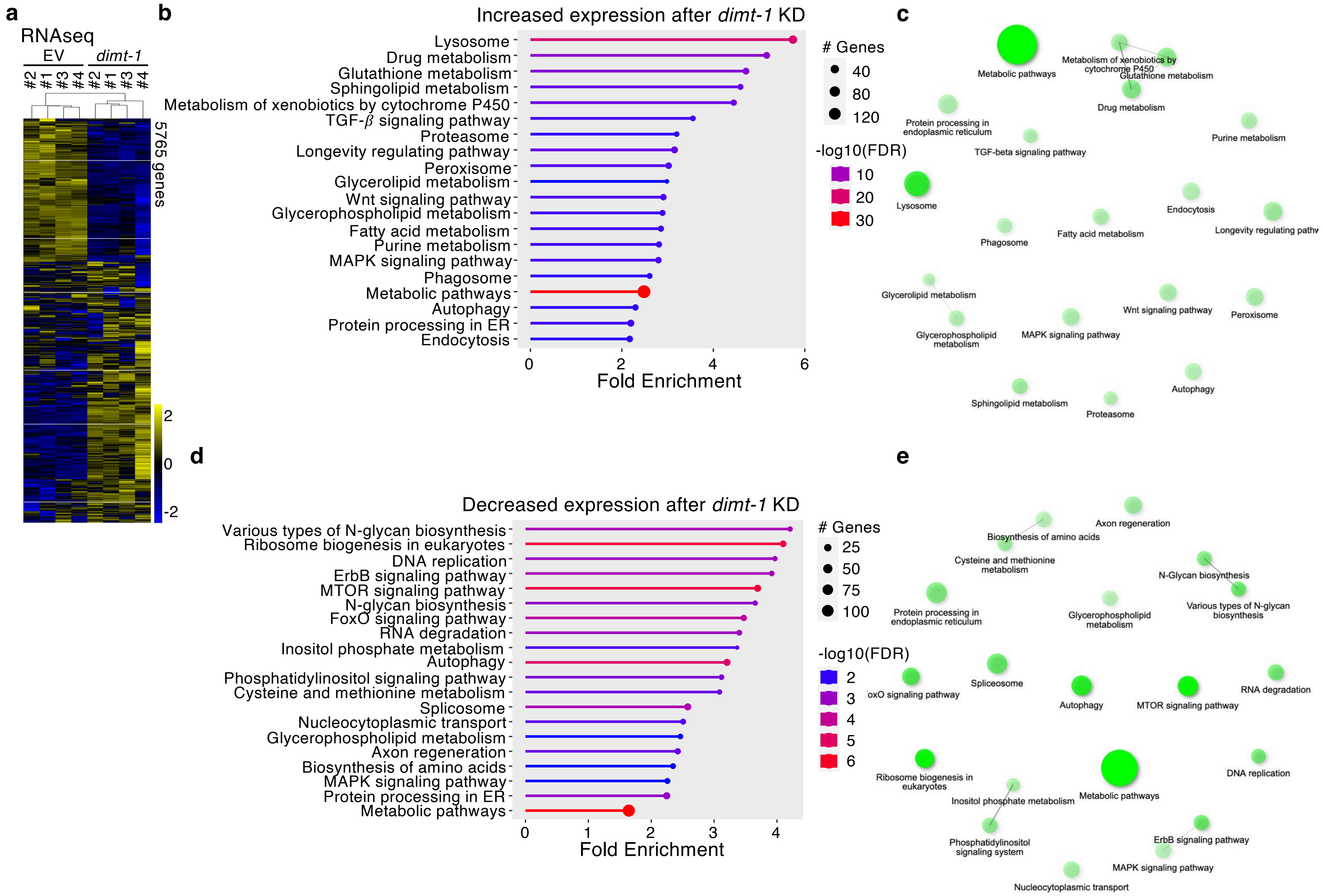

Rothi MH, Sarkar GC, Al Hadad J et al Fig S4

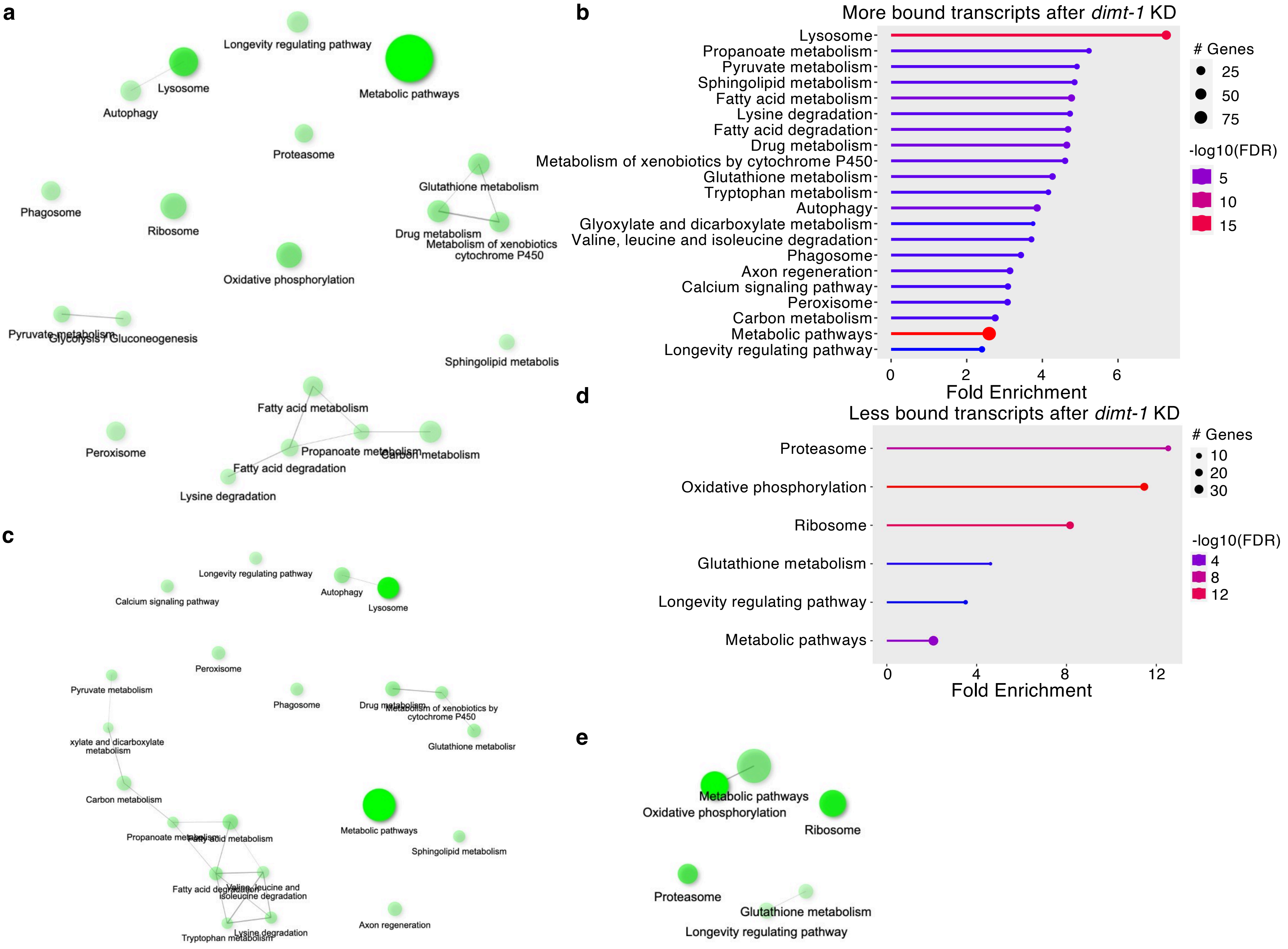

Rothi MH, Sarkar GC, Al Hadad J et al Fig S5

Strain	RNAi	Mean +/- SEM	Median	p values	# worms	Figure
WT	EV	16.211 +/- 0.3620	17		128/163	3E
WT	*dimt-1*	17.949 +/- 0.4724	17	0.0012	117/172	3E
hsf-1	EV	14.714 +/- 0.5969	15		49/70	3E
hsf-1	*dimt-1*	17.951 +/- 0.5677	17	0.0031	68/87	3E
daf-16(mu86)	EV	15.279 +/- 0.4190	15		68/82	
daf-16(mu86)	*dimt-1*	15.921 +/- 0.2927	17	0.3626	63/90	
eat-2(ad1116)	EV	23.433 +/- 1.318	24		60/88	
eat-2(ad1116)	*dimt-1*	26.286 +/- 1.249	28	0.6196	35/91	
glp-1(e2141ts)	EV	21.258 +/- 0.6080	21		89/91	
glp-1(e2141ts)	*dimt-1*	22.943 +/- 0.7214	24	0.0189	88/95	
WT	EV	17.967 +/- 0.3376	19		90/92	
WT	*dimt-1*	20.208 +/- 0.4085	21	<0.0001	77/88	
daf-16(mu86)	EV	16.5 +/- 0.3545	17		88/88	
daf-16(mu86)	*dimt-1*	18.053 +/- 0.3685	19	0.0017	75/79	
eat-2(ad1116)	EV	23.728 +/- 0.6403	21		92/96	
eat-2(ad1116)	*dimt-1*	25.639 +/- 0.6912	26	0.0574	83/87	
raga-1	EV	27.242 +/- 0.7933	28		95/95	
raga-1	*dimt-1*	26.659 +/- 0.7021	26	0.5407	86/87	
daf-2(e1370)	EV	38.029 +/- 1.435	41		70/97	
daf-2(e1370)	*dimt-1*	48.75 +/- 2.028	51	<0.0001	61/94	
glp-1(e2141ts)	EV	26.69 +/- 0.7349	28		100/102	
glp-1(e2141ts)	*dimt-1*	22.553 +/- 0.5352	23	<0.0001	103/104	
WT	EV	17.414 +/- 0.3643	17		87/96	3C,3F,S3A
WT	*dimt-1*	21.533 +/- 0.4659	21	<0.0001	75/94	3C,3F,S3A
daf-16(mu86)	EV	15.230 +/- 0.3720	15	0.0001	87/96	3F
daf-16(mu86)	*dimt-1*	16.012 +/- 0.4390	15	0.0907	81/96	3F
eat-2(ad1116)	EV	20.986 +/- 0.7766	7	<0.0001	72/96	3C
eat-2(ad1116)	*dimt-1*	27.397 +/- 0.5261	19	<0.0001	68/74	3C
hsf-1	EV	7.8 +/- 0.6208	19	<0.0001	45/76	
hsf-1	*dimt-1*	17.375 +/- 0.6098	27	<0.0001	59/66	
clk-1	EV	19.107 +/- 0.6696	19	0.0042	75/96	S3A
clk-1	*dimt-1*	25.833 +/- 0.7145	27	<0.0001	78/96	S3A
WT	EV	15.181 +/- 0.4120	14		94/98	3H
WT	*dimt-1*	19.481 +/- 0.5768	20	<0.0001	81/91	3H
glp-1(e2141ts)	EV	26.259 +/- 0.6842	26	<0.0001	81/82	3H
glp-1(e2141ts)	*dimt-1*	24.885 +/- 0.7855	24	0.2013	61/73	3H
pgl-1	EV	18.443 +/- 0.9143	16	<0.0001	97/98	
pgl-1	*dimt-1*	17.674 +/- 0.7517	18	0.2133	92/97	
WT	EV	17.90 +/- 0.2304	18		83/96	
WT	*dimt-1*	21.64 +/- 0.4192	23	<0.0001	81/96	
WT	EV	19.45 +/- 0.4091	21		65/91	
WT	*dimt-1*	26.86 +/- 1.269	24	0.0003	79/89	
raga-1	EV	27.60 +/- 0.9856	31	<0.0001	43/88	
raga-1	*dimt-1*	30.03 +/- 1.260	27	0.4212	65/89	

Supplementary Table 1. Dimt-1 depletion extends lifespan in a raga-1 and germline dependent manner The figure panels in which specific experiments are shown or used are indicated in the right column. The mean lifespan and SD values were calculated by Prism from triplicate samples of 30 worms each (90 worms total). # worms: number of observed dead worms at the end of the experiment/number of alive worms at the beginning of the experiment. The difference between both numbers corresponds to the number of censored worms (worms that underwent “matricide”, exhibited ruptured vulva, or crawled off the plates). P values are calculated by log rank (Mantel-Cox) statistical test.

Strain	RNAi	Mean +/- SD	Median	p values	# worms	Figure
WT	EV	17.778 +/- 0.4064	17		90/94	3D, 3I
WT	*dimt-1*	24.918 +/- 0.6084	27	<0.0001	50/60	3D, 3I
pgl-1	EV	18.449 +/- 0.4319	20	0.2051	99/99	3I
pgl-1	*dimt-1*	19.065 +/- 0.4880	20	0.2807	94/94	3I
daf-2(e1370)	EV	38.548 +/- 1.974	41	<0.0001	31/45	3D
daf-2(e1370)	*dimt-1*	51.647 +/- 2.980	55	<0.0001	34/68	3D
WT FUdR	EV	17.581 +/- 0.3725	19		83/92	
WT FUdR	*dimt-1*	15.709 +/- 0.3695	17	<0.0001	87/94	
WT FUdR	EV	17.953 +/- 0.2396	18		85/92	4E
WT FUdR	*dimt-1*	17.400 +/- 0.2452	18	0.0773	90/93	4E
WT	EV	16.281 +/- 0.5470	15		90/92	3G, 5D
WT	*dimt-1*	19.971 +/- 0.9724	20	<0.0001	77/88	3G, 5D
daf-16(mu86)	EV	12.943 +/- 0.7447	13		88/88	
daf-16(mu86)	*dimt-1*	12.964 +/- 0.7372	13	0.9641	75/79	
daf-9(rh50)	EV	17.88 +/- 0.7043	18		92/96	5D
daf-9(rh50)	*dimt-1*	17.193 +/- 0.7995	18	0.5511	83/87	5D
raga-1	EV	24.402 +/- 0.7772	22		95/95	3G
raga-1	*dimt-1*	24.531 +/- 0.7260	22	0.7892	86/87	3G
WT	EV	14.51 +/- 0.42			67/90	5E
WT	*dimt-1*	16.98 +/- 0.52		0.0002	94/94	5E
daf-12(rh61rh412)	EV	12.42 +/- 0.20			96/96	5E
daf-12(rh61rh412)	*dimt-1*	12.28 +/- 0.20		0.57	81/90	5E
daf-9(rh50)	EV	13.22 +/- 0.19			94/90	
daf-9(rh50)	*dimt-1*	12.84 +/- 0.22		0.34	80/90	
WT	EV	16.43 +/- 0.58			76/90	
WT	*dimt-1*	19.31 +/- 0.45		<0.0001	140/140	
daf-12(rh61rh412)	EV	13.74 +/- 0.46			79/90	
daf-12(rh61rh412)	*dimt-1*	13.86 +/- 0.47		0.89	74/90	
daf-9(rh50)	EV	13.7 +/- 0.46			65/90	
daf-9(rh50)	*dimt-1*	13.53 +/- 0.38		0.87	102/102	
WT		15.512 +/- 1.992	15		83/96	3B
dimt-1 E79A		21.637 +/- 0.2514	22	<0.0001	81/96	3B
WT		15.383 +/- 1.899	15		83/96	
dimt-1 E79A		21.3 +/- 0.2638	20	<0.0001	81/96	
WT		15.598 +/- 1.703	15		83/96	
dimt-1 E79A		20.883 +/- 0.3052	20	<0.0001	81/96	

Supplementary Table 1. Dimt-1 depletion extends lifespan in a raga-1 and germline dependent manner The figure panels in which specific experiments are shown or used are indicated in the right column. The mean lifespan and SD values were calculated by Prism from triplicate samples of 30 worms each (90 worms total). # worms: number of observed dead worms at the end of the experiment/number of alive worms at the beginning of the experiment. The difference between both numbers corresponds to the number of censored worms (worms that underwent “matricide”, exhibited ruptured vulva, or crawled off the plates). P values are calculated by log rank (Mantel-Cox) statistical test.

Strain	TIR1	Time Placed on auxin	Mean +/- SD	Median	p values	# worms	Figure
WT		P-1 L4	16.233 +/- 0.4988	16	0.0746	60/93	
dimt-1::AID		P-1 L4	17.123 +/- 0.6470	16		73/96	4A
dimt-1::AID	CA1200 eft-3 ubiquitous	P-1 L4	22.211 +/- 0.8067	22	<0.0001	57/85	4A
dimt-1::AID	DV3801 unc-54 muscle	P-1 L4	17.36 +/- 0.6112	17	0.9993	75/92	4A
dimt-1::AID	DV3803 ges-1 intestine	P-1 L4	17.922 +/- 0.6650	18	0.5125	51/88	4A
dimt-1::AID	DV3805 rgef-1 neuron	P-1 L4	17.85 +/- 0.6920	18	0.6655	40/75	4A
dimt-1::AID	JDW221 mex-5 germline	P-1 L4	25.351 +/- 0.8675	26	<0.0001	74/97	4A
WT		P-1 L4	16.86 +/- 0.4803	17		71/90	
dimt-1::AID		P-1 L4	18.66 +/- 0.6840	19		76/90	S2B
dimt-1::AID	CA1200 eft-3 ubiquitous	P-1 L4	21.03 +/- 0.7266	21	0.0007	74/103	S2B
dimt-1::AID	DV3801 unc-54 muscle	P-1 L4	17.31 +/- 0.6508	17	0.2456	72/94	
dimt-1::AID	DV3803 ges-1 intestine	P-1 L4	17.17 +/- 0.7407	17	0.2780	69/91	
dimt-1::AID	DV3805 rgef-1 neuron	P-1 L4	17.34 +/- 0.6514	15	0.1238	77/95	
dimt-1::AID	JDW221 mex-5 germline	P-1 L4	24.20 +/- 0.8902	25	<0.0001	70/73	S2B
dimt-1::AID	JDW225 eft-3 ubiquitous	egg	24.42 +/- 0.7870	25	<0.0001	52/18	S2B

Strain	RNAi	Mean +/- SD	Median	p values	# worms	Figure
WT	EV	15.063 +/- 0.6362	15		64/90	4C
WT	*dimt-1*	19.433 +/- 0.5664	20	<0.0001	67/90	4C
NR350	EV	17.326 +/- 0.6276	17		43/90	4C
NR350	*dimt-1*	16.95 +/- 0.7071	17	0.8636	40/90	4C
DCL569	EV	16.222 +/- 0.5706	15		72/90	
DCL569	*dimt-1*	17.649 +/- 0.5898	17	0.0924	74/90	
IG1836	EV	14.2 +/- 0.3896	13		70/98	4C
IG1836	*dimt-1*	14.768 +/- 0.4562	15	0.2763	69/90	4C
XE14781	EV	12.086 +/- 0.4714	10		58/90	
XE14781	*dimt-1*	12.413 +/- 0.3590	13	0.6165	63/90	
WT	EV	17.170 +/- 0.7227	17		53/90	4D
WT	*dimt-1*	19.984 +/- 0.6028	20	0.0134	64/90	4D
NR350	EV	16.923 +/- 0.7433	17		39/90	
NR350	*dimt-1*	18.412 +/- 0.6861	18.5	0.2357	34/90	
DCL569	EV	16.347 +/-0.4772	15		72/90	4D
DCL569	*dimt-1*	18.951 +/- 0.4957	20	0.0004	82/90	4D
IG1836	EV	15.226 +/- 0.4148	15		62/90	
IG1836	*dimt-1*	14.761 +/- 0.4563	13	0.4794	71/90	
XE14781	EV	11.952 +/- 0.3383	13		62/90	4D
XE14781	*dimt-1*	12.412 +/- 0.3859	13	0.2677	68/90	4D
EV484	EV	18.30 +/- 0.25	18		47/53	
EV484	*dimt-1*	19.10 +/- 0.46	20	0.0141	58/67	
EV484	EV	18.38 +/- 0.27	19		76/85	
EV484	*dimt-1*	17.58 +/- 0.37	18	0.1787	54/60	
EV484	EV	16.49 +/- 0.30	17		49/54	
EV484	*dimt-1*	18.82 +/- 0.33	19	<0.0001	49/53	
EV484	EV	17.85 +/- 0.24	18		62/67	
EV484	*dimt-1*	19.75 +/- 0.40	20	<0.0001	40/46	
EV484	EV	17.24 +/- 0.25	17		98/107	S2C
EV484	*dimt-1*	19.48 +/- 0.38	20	<0.0001	63/65	S2C

Supplementary Table 2. DIMT-1 functions in the germline to regulate lifespan The figure panels in which specific experiments are shown or used are indicated in the right column. The mean lifespan and SD values were calculated by Prism from triplicate samples of 30 worms each (90 worms total). # worms: number of observed dead worms at the end of the experiment/number of alive worms at the beginning of the experiment. The difference between both numbers corresponds to the number of censored worms (worms that underwent “matricide”, exhibited ruptured vulva, or crawled off the plates). P values are calculated by log rank (Mantel-Cox) statistical test.

Strain	TIR1	Time Placed on auxin	Mean +/- SD	Median	p values	# worms	Figure
dimt-1::AID	JDW221 mex-5 germline		14.127 +/- 0.4027	14		72/90	
dimt-1::AID	JDW221 mex-5 germline	P-1 L4	21.5 +/- 0.5959	22	<0.0001	80/90	5B
dimt-1::AID	JDW221 mex-5 germline	egg	19.966 +/- 0.5115	20	<0.0001	89/93	5B
dimt-1::AID	JDW221 mex-5 germline	egg->y.a.	14.195 +/- 0.2814	14	0.9570	83/94	5B
dimt-1::AID	JDW221 mex-5 germline	y.a.	19.519 +/- 0.4576	22	<0.0001	75/87	5B
dimt-1::AID	CA1200 eft-3 ubiquitous		14.152 +/- 0.3988	14		67/90	5C
dimt-1::AID	CA1200 eft-3 ubiquitous	P-1 L4	20.159 +/- 0.5473	22	<0.0001	69/93	5C
dimt-1::AID	CA1200 eft-3 ubiquitous	egg	18.464 +/- 0.6297	20	<0.0001	69/90	5C
dimt-1::AID	CA1200 eft-3 ubiquitous	egg->y.a.	15.588 +/- 0.5234	16	0.0101	68/91	5C
dimt-1::AID	CA1200 eft-3 ubiquitous	y.a.	19.169 +/- 0.6477	22	<0.0001	66/95	5C
dimt-1::AID	JDW221 mex-5 germline		13.96 +/- 0.4276	14		53/94	
dimt-1::AID	JDW221 mex-5 germline	P-1 L4	21.74 +/- 0.9826	19	<0.0001	68/91	
dimt-1::AID	JDW221 mex-5 germline	egg	20.78 +/- 0.7348	21	<0.0001	85/94	
dimt-1::AID	JDW221 mex-5 germline	egg->y.a.	15.21 +/- 0.4364	14	0.1426	66/80	
dimt-1::AID	JDW221 mex-5 germline	y.a.	19.69 +/- 0.8134	21	<0.0001	64/90	
dimt-1::AID	CA1200 eft-3 ubiquitous		13.93 +/- 0.5767	14		42/87	
dimt-1::AID	CA1200 eft-3 ubiquitous	P-1 L4	17.79 +/- 0.7352	19	<0.0001	61/101	
dimt-1::AID	CA1200 eft-3 ubiquitous	egg	17.82 +/- 0.8017	19	<0.0001	65/96	
dimt-1::AID	CA1200 eft-3 ubiquitous	egg->y.a.	15.35 +/- 0.7266	16	0.0890	63/96	
dimt-1::AID	CA1200 eft-3 ubiquitous	y.a.	17.18 +/- 0.7952	16	0.0004	55/92	
dimt-1::AID	JDW221 mex-5 germline		18.24 +/- 0.4745	17		75/90	
dimt-1::AID	JDW221 mex-5 germline	y.a.	21.182 +/- 0.5970	22	<0.0001	77/90	
dimt-1::AID	JDW221 mex-5 germline	PEL	22.829 +/- 0.6534	22	<0.0001	70/90	
dimt-1::AID	JDW221 mex-5 germline	ML	21.227 +/- 0.6159	22	0.0002	66/90	
dimt-1::AID	JDW221 mex-5 germline		18.136 +/- 0.4999	19		81/91	5D
dimt-1::AID	JDW221 mex-5 germline	y.a.	22.056 +/- 0.6425	21	<0.0001	89/91	5D
dimt-1::AID	JDW221 mex-5 germline	PEL	22.671 +/- 0.6702	23	<0.0001	70/90	5D
dimt-1::AID	JDW221 mex-5 germline	ML	21.569 +/- 0.6783	21	<0.0001	65/90	5D

Supplementary Table 5. DIMT-1 functions after mid-life to regulate lifespan The figure panels in which specific experiments are shown or used are indicated in the right column. The mean lifespan and SD values were calculated by Prism from triplicate samples of 30 worms each (90 worms total). # worms: number of observed dead worms at the end of the experiment/number of alive worms at the beginning of the experiment. The difference between both numbers corresponds to the number of censored worms (worms that underwent “matricide”, exhibited ruptured vulva, or crawled off the plates). P values are calculated by log rank (Mantel-Cox) statistical test.
